# Degree of Acceptance of Virtual Reality by Health Sciences Students

**DOI:** 10.3390/ijerph20085571

**Published:** 2023-04-18

**Authors:** Julio Cabero-Almenara, Carmen Llorente-Cejudo, Antonio Palacios-Rodríguez, Óscar Gallego-Pérez

**Affiliations:** 1 Department of Didactics and Educational Organization, University of Seville, 41013 Seville, Spain; 2 Department of Audiovisual Media and New Technologies Service, University of Seville, 41013 Seville, Spain

**Keywords:** TAM, degree of acceptance of technology, university education, virtual reality, health sciences

## Abstract

Virtual Reality (VR) is an emerging technology with educational benefits demonstrated in numerous studies. Its integration into the curriculum implies the use of cognitive resources by students and the training of digital skills by teachers. The objective of this study is to determine the degree of acceptance of students with learning objects produced in VR and in 360°, as well as to analyze their evaluation and the established relationships. A sample of 136 medical students who completed questionnaires on the Technology Acceptance Model (TAM) and the quality of the training activity was used. The results show high levels of acceptance, both in VR and 360° objects. The students perceived the usefulness of the training activity as high, with significant correlations between the different dimensions. This study demonstrates the potential of VR as an educational technology and offers new perspectives for future research.

## 1. Introduction

Virtual Reality [1,2,3,4,5] is a technology presented in various reports prepared to demonstrate the technological trends that will be incorporated into teaching in the near future, alone or interacting with other technologies, such as “Artificial Intelligence” or “e-learning”. When relating it to other technologies, such as augmented technology, some studies call attention to the fact that in virtual reality, “virtual data replaces physical data, creating a new reality”.

Likewise, it is also highlighted that, with it, “an environment that may or may not appear real is created, which gives the user the feeling of being immersed in it”. It is this sensation that can be useful to promote learning through simulation or experiential learning [6].

In order to specify the terms we are using, the differences between Augmented Reality (AR) and Virtual Reality (VR) were established [7] based on a series of differences that are specified in Table 1.

It should also be noted that within VR, we find two types: immersive and non-immersive [8,9]. The first has the following characteristics: high cost, its use is complex, it tends to cause disorientation in the person, it offers a great sensation of reality, and it provides a sensation of total immersion; the second offers a more accessible cost, is easier to use, offers quick acceptance, expresses a lesser sense of reality, and partial immersion.

The interest aroused by the educational use of VR can be seen in the increase in its research. This is evidenced by the evolution in the performance of meta-analyses on the studies carried out [10,11,12,13,14]. These studies generally show that it is a technology that offers real educational possibilities.

Although there were errors at the beginning, we insist that this technology will not disappear and that it is, therefore, necessary to increase experimentation and research on it.

Based on these comments, we believe it is also necessary to propose models on which we can rely to facilitate the design of their learning objects and proposals for use, and in this sense, [15] alludes to problem-based, experiential, and collaborative learning, that is, by creating training scenarios that promote student learning through reflection on the activities carried out [16,17,18,19] and in more active and participatory scenarios [13].

On the other hand, different investigations have shown that its use increases the motivation of students towards the task and the contents that they must learn [19,20,21,22], as well as use improvements in learning in general [11,23,24], and it is useful for improving the competence of classroom climate management [25].

Its recent expansion in training contexts is due to two fundamental reasons: the power that computers are acquiring to present quality audiovisual and multimedia information offered through these resources and a decrease in the cost of equipment.

Finally, it should be noted that it is the field of health sciences where VR is being used the most. Virtual reality (VR) technology has become increasingly popular in the field of medicine due to its numerous benefits [26]. Here are some of the benefits of virtual reality for the study of medicine:

1. Enhanced learning experience: With VR, medical students and healthcare professionals can have a more immersive learning experience. They can visualize and interact with anatomical structures and medical scenarios in a way that was not possible before, leading to a more engaging and effective learning experience.
2. Realistic simulations: VR allows for the creation of realistic simulations of medical procedures and scenarios, providing a safe and controlled environment for students to practice and improve their skills without risk to real patients.
3. Increased patient safety: The use of VR can improve patient safety by allowing medical professionals to practice complex procedures and surgeries before performing them on real patients.
4. Cost effective: Traditional medical training can be expensive due to the need for specialized equipment and facilities. VR provides a cost-effective alternative to traditional training, as it eliminates the need for expensive equipment and reduces the need for training with live patients.
5. Collaborative learning: VR can facilitate collaborative learning among medical professionals, allowing them to work together on simulations and learn from each other’s experiences and feedback.
6. Overall, virtual reality technology has the potential to revolutionize medical education and improve patient care, making it an exciting development in the field of medicine.

Presenting itself effectively to be used through simulations, which is one of the most used teaching strategies in these areas of knowledge, in different processes and contexts, facilitates the student’s approach to reality [3,23,27,28,29,30]. Specifically, in nursing, it has been one of the tools traditionally used [31,32].

### The Adoption towards the Use of Technologies by Students

In 1989, Davis presented a model with which he suggests that the beliefs that a person has about the consequences of using a type of technology will determine the use that he/she makes of it. He calls this the “Technology Acceptance Model”, or TAM as it is usually known [33].

**Figure 1:**
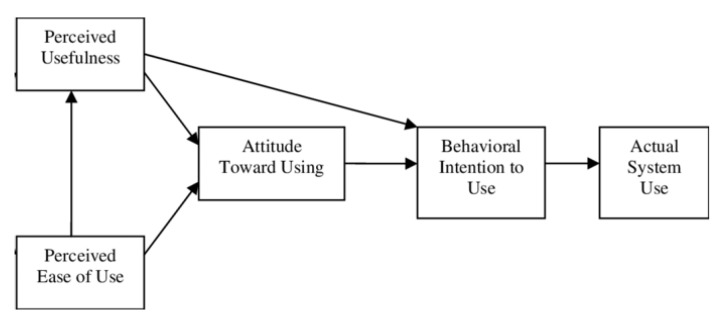
TAM model.

This model indicates that the attitude or predisposition that one has regarding the intention to use technology is determined by the perceived usefulness (Perceived Usefulness) and the perceived ease of use (Perceived Ease of Use), configuring the model offered in Figure 1.

This model has been adapted to new proposals, such as TAM2 [34], TAM3 [35], or the so-called “Unified Theory of Technology Acceptance” (UTAUT) [35], that add new variables to explain the acceptance of a given technology by people; such changes do not radically transform the original TAM model. Let us point out that the present study will use the original model. By examining the perceived usefulness and perceived ease of use of these technologies among medical professionals, researchers can gain insight into the factors that influence their acceptance and adoption.

Different studies and meta-analyses of research carried out where the TAM model was applied [36,37] have indicated that it is a valid and robust model to explain the intention to use any technology and that it is easy to apply and analyze.

With it, research has been carried out on different technologies, such as m-learning [38], e-learning [39], computer games [40], mobile devices [41], or augmented reality [42].

Based on what has been discussed, this study aims to find out the degree of acceptance of VR technology and 360° video among health sciences students. We also intended to study the relationships with student satisfaction.

## 2. Materials and Methods

### 2.1. Hypothesis and Research Objectives

In our research, different hypotheses are proposed, some of which derive directly from the TAM model. The hypotheses formulated were according to [39]:

- H1-H2-H3: The perception of ease of use can positively and significantly affect the perception of enjoyment, the perceived usefulness, and the attitudes toward the use of the learning objects produced in VR.
- H4-H5-H6: The perceived utility of the use of learning objects in VR can positively and significantly affect, with respect to the perception of enjoyment, the attitude towards the use and the intentions of using the objects in VR.
- H7-H8: The perception of enjoyment can positively and significantly affect attitudes towards the use of VR learning objects and intentions to use VR objects.
- H9: The attitude towards the use can positively and significantly affect the intention to use learning objects in VR.

We also wanted to know if there were significant relationships between the different dimensions with the total score obtained in the TAM.

Finally, we analyzed whether the evaluation made by the students of the different objects was related to the degree of satisfaction shown by the students of the experience developed in VR.

### 2.2. Sample

The 2021–2022 academic year was the one in which the experience was developed, and 136 students participated in it and were enrolled in the subject “Fundamentals in Surgery and Anesthesiology” taught as part of the Health Sciences Degree at the University of Seville. The vast majority of students enrolled in the subject participated in the research; therefore, the sampling can be understood as convenience or causal [43].

In addition, all participants were informed about the objectives of this study, and all the participants signed an informed consent and data release clause.

### 2.3. Produced Objects

The objective pursued by the elaborated materials was to facilitate one of those that medical students should pursue within the area of knowledge of surgery, that the student must carry out practices in the surgical area in order to become familiar with them, and is not only with surgical techniques and skills but also learning to function in an environment that is very hostile and demanding for those who are not used to it.

Added to this is the fact that third-year students have not been admitted to the hospital for internships up to that moment, and they are facing a rotation for the first time, which can be stressful in many cases.

That is why it would be interesting to try to reduce the stress of this experience by previously showing the student the scenario that they will encounter in future practices, so that they are more secure and oriented.

The three scenarios that the student will face are surgery consultations, the area known as cleaning where handwashing is carried out prior to surgery, and, finally, inside the operating room.

For this reason, we created three learning objects in virtual reality format and incorporated 360° videos in them. In order to explain these three areas that they will have to face in the practices: how to behave in the consultation with the patient in the communication of the diagnosis, the action of washing hands before surgery, and how and where one can move within an operating room, it should be noted that the three objects produced can be classified from [9] as non-immersive.

In the following links, the reader can access the observation of the three learning objects produced:

- https://bit.ly/3F0M57n (accessed on 23 February 2023)
- https://bit.ly/3JfKgpl (accessed on 23 February 2023)
- https://bit.ly/3EZd17t (accessed on 23 February 2023)

All the objects began with a poster in which the student was shown the devices through which they could observe the objects produced and offered a brief description of the information that was going to be presented in the process. Let us say, from the beginning, that the materials were designed under two principles: mono-conceptual, and experiential and reality simulators. It should be noted that “hot spots” were incorporated into the objects that provided additional information regarding the learning that students should acquire or the specifications of what was being carried out through video clips or specific images.

The number of “hot spots” and the type of information were different in each of the objects; Table 2 shows a list of these.

It should be noted that only one of the objects (consultation) included sounds, which referred to the comments between the doctor and the patient.

Additionally, in the objects in the process of finishing the interaction, an informative poster appeared for the student to complete a questionnaire that allowed us to know if they had learned the information that had been offered in the different objects produced.

The realization of the different objects was carried out using the following resources and programs: 360 one R camera, the editing software offered by the camera, the Premiere software for video editing, and the KRPANO program for creating the “hot spots”. Finally, the programs were uploaded to a server.

The students were provided with the aforementioned URLs, and their observation was carried out in the classroom context to interact with the objects produced, completing the questionnaires at the end of each interaction, as indicated above.

The total duration of the experience was 2 weeks, including: contact, experimentation, and completion of the questionnaires.

### 2.4. Instruments

There were two instruments for collecting information. In the case of the one used for the analysis of the TAM, it should be noted that the basic structure of its creator [33] was followed for the degree of acceptance of RA technology. The instrument collects information from the four dimensions of the TAM model: perceived usefulness (UP), perceived ease of use (FUP), perceived enjoyment (DP), attitude towards use (AU), and intention to use it (UI); it was made up of 15 Likert-type items, with seven response options ranging from 1 = Extremely unlikely/disagree to 7 = Extremely likely/agree.

In the case of the one referring to the evaluation of the technical quality of the objects produced in VR, it was an “ad hoc” questionnaire with a Likert-type construction, made up of 13 items with 6 response options (MP = Very positive/Strongly agree (6); P = Positive/Agree; R+ = Regularly positive/Moderately in agreement; R− = Regularly negative/Moderately in disagreement; N = Negative/Disagree; and MN = Very negative/strongly in disagreement (1)), which sought to collect information on three dimensions: technical and aesthetic aspects (4 items), ease of use (7 items), and evaluation of the guide prepared to explain the operation of the object, developed for the evaluation of AR objects [42,44,45]. All the instruments were administered via the Internet.

## 3. Results

Prior to presenting the results, the reliability indices of the questionnaires are shown. The first questionnaire (TAM) obtains a Cronbach’s Alpha index of 0.978 and the second (satisfaction) an index of 0.967. Both indices are considered excellent according to previous literature [44].

The degree of acceptance of the different objects produced was quite high since, in the entire instrument used, the average obtained was 6,108, on a scale of 1 to 7, and with a very low standard deviation of 0.834, which indicates a certain homogeneity of the scores offered by the students.

Regarding the general dimensions that make up the TAM model, the mean scores and standard deviations achieved are presented in Table 3.

As we can see, the average scores are around the value 6, with which the students’ evaluations focused on the answer: “quite probable/agree”. At the same time, it should be noted that the scores were very similar in the five dimensions, with an average score of 6.38 standing out from all of them referring to “Intention to use”.

Table 4 shows the mean values and standard deviations achieved in each of the items of the instrument.

For the analysis of some of the hypotheses that emerge from the TAM model formulated for this research, specifically those referring to the significance of the large dimensions, we apply Spearman’s correlation coefficient (Table 5).

On the other hand, when the relationship between the TAM score achieved and the different dimensions that make up the instrument was analyzed, the scores achieved are presented in Table 6.

The results achieved allow us to point out, on the one hand, the reliability of the model, since the relationships between all its dimensions and the dimensions with the total obtained in the instrument are significant, positive, and high. Then, it can be pointed out that there is a good fit in the model used.

On the other hand, seeing the correlations between the different dimensions, different conclusions are established: (1) direct, positive, and significant relationships were found between the different contrasted dimensions of the TAM, so that when one of them increases, so does the other; (2) all relationships are at *p* ≤ 0.01; and (3) the found relationships range from moderate to high.

Specifically, it is noted that:

- The perception of ease of use positively and significantly affects the perception of enjoyment, the perceived usefulness, and the attitudes of use towards the learning objects produced in VR (H1-H2-H3).
- The perceived usefulness of the use of learning objects in VR positively and significantly affects, regarding the perception of enjoyment, the attitude towards the use, in the intentions of using the objects in VR (H4-H5-H6).
- The perception of enjoyment positively and significantly affects attitudes towards the use of learning objects in VR and intentions to use the objects in VR (H7-H8).
- The attitude towards the use positively and significantly affects the intention to use learning objects in VR (H9).

With regard to the evaluation carried out by the students of the different objects, the average scores achieved in the three dimensions and in the overall instrument are presented in Table 7.

As we can see, the average scores achieved, on a scale ranging from 1 (MN = Very negative/strongly disagree) to 6 (MP = Very positive/strongly agree), were high, and, therefore, we could say that the objects were perceived as useful, easy to handle, and aesthetically attractive.

Finally, we wanted to know if there were relationships between the evaluations made of the objects produced in VR and the degree of acceptance shown by the students. For this, we applied Spearman’s correlation coefficient again. The values found are presented below, both with the total of the TAM instrument and with its different dimensions (Table 8).

## 4. Discussion

One of the objectives of our study was to observe the degree of acceptance that students had whilst participating in an experience with learning objects made in VR, non-immersive, and with 360° recordings. In this regard, it should be noted that the technology used and, more specifically, its design have aroused a high degree of acceptance by students. This leads us to suggest that it is a significant technology to be applied to the training of these future professionals, as has already been found in other investigations where they were used in the training of doctors [3,23,27,28,29].

At the same time, it becomes clear that VR learning objects, designed and produced under a monothematic orientation, with the inclusion of informative and indicative “hot spots” and with a tendency towards multimedia production, arouse a high level of interest and degree of acceptance in students and are valued very positively by them.

In the investigation, we also found that the model used [33] is valid and robust, for the explanation of the tendency to use VR by students.

## 5. Conclusions

Our study leads to the conclusion that students showed a high degree of acceptance for VR, showing a strong attitude towards its use in training, indicating a strong intention to use it in the future. This leads us to the same opinion as other authors [46] who indicate that we must study the preferences that students have towards technologies, which is, in this case, VR, for its incorporation into training scenarios.

This assessment also reinforces that the students endorsed the quality of the objects produced and their form of design, which was positive, both globally and for the different dimensions that made up the evaluation instrument used.

The evaluation that the students made of the objects as their degree of acceptance showed a significant and positive relationship.

As has been proposed by various authors [47,48,49,50], it is worth pointing out for future lines of research to analyze its use in new emerging pedagogies, such as “flipped learning”, or to analyze the degree of acceptance with other formulated models, such as the UTAUT, regarding which a series of works are already being carried out [51].

This study highlights the positive attitudes that students have towards the use of virtual reality (VR) in their training. The results show that students had a high level of acceptance of VR and were strongly inclined towards using it in the future. This is an important finding as it suggests that VR technology has the potential to be an effective tool for enhancing student learning experiences.

Furthermore, the study reinforces the need for educators to understand students’ preferences towards technology, specifically VR, to effectively incorporate it into training scenarios. This approach is consistent with the recommendations of other researchers who also emphasized the importance of studying student preferences and attitudes towards technology when integrating it into educational settings [52].

Overall, the study’s findings highlight the potential of VR technology in enhancing the learning experiences of students, and the importance of considering students’ preferences when incorporating technology into training scenarios. By doing so, educators can develop effective strategies for using VR to facilitate student learning and engagement in the classroom [53].

As limitations of the research, the non-random selection of students must be assumed, having worked with natural groups, and not having controlled the “novelty effect” in exposure to technologies. In addition, the sample size was reduced. This is due to access to the sample at the university where the experience was carried out. Even so, the sample is representative of the reality where the research was carried out. For future studies, it is proposed to replicate both the experience and the type of study in other related contexts.

## Figures and Tables

**Table 1:** Differences between AR and VR.

Characteristic	Augmented Reality	Virtual Reality
Role of the local physical environment	Extends/decreases	Is replaced
Usage time frame (potential)	Durable	Temporarily
Typical usage context	Everywhere	In a “safe” area (e.g., at home) or in specific contexts (e.g., therapy, amusement parks, stores, etc.).
Technology	Devices: Stationary, mobile, portable, on the body, projectors. Visualization techniques: transparent video screens Optical transparent screens. Projection.	Devices: Wearables (HMD), caves (decreasing practical relevance). Visualization techniques: video screens, projection.
Physical risks	Collisions or accidents due to distraction.	Collisions or accidents due to disconnection with the real world.
Privacy concerns	The user and the people around him.	The user
Dizziness	Rarely applicable	Important
Specific mechanism	Local presence	Telepresence
Typical use cases	Situations where combined experiences of real and virtual content are beneficial (e.g., to compare sizes, e.g., of furniture) and possible (e.g., the home for furniture already exists).	Situations where the physical or story context does not exist (e.g., a fictional game), is not accessible to a user (e.g., the moon, time travel), or where the actual physical context does not is desirable (e.g., in training situations that would be dangerous in the real world).

**Table 2:** Resources embedded in the three objects produced.

	Operating Room	Handwashing	Query
Informational Hot Spots	14 (typology of presentation and description).	5 (information expansion typology).	2 (one of evolution and with five informative posters calling for attention and one informative).
Video clips	1		
Location zone points	2	5	2

**Table 3:** Means and standard deviations in the different dimensions of the TAM.

Dimension	M	SD
Perceived Utility (UP)	5.90	1.01
Perceived ease of use (FUP)	6.04	1.01
Perception of Enjoyment (DP)	6.05	1.10
Attitude towards use (AU)	6.17	0.939
Intent to Use (IU)	6.38	0.912

**Table 4:** Mean and standard deviation of each of the items.

	M	SD
The use of this VR system will improve my learning and performance in this subject (UP1)	5.63	1.288
Using the VR system during classes would make it easier for me to understand certain concepts (UP2)	5.94	1.134
I think the VR system is useful when learning (UP3)	6.13	1.022
Using VR would increase my performance (UP4)	5.89	1.127
I think the VR system is easy to use (FUP1)	5.86	1.248
Learning to use the VR system is not a problem for me (FUP2)	6.15	1.108
Learning to use the VR system is clear and understandable (FUP3)	6.10	1.077
Using the VR system is fun (DP1)	6.07	1.206
I enjoyed using the VR system (DP2)	5.93	1.263
I think the VR system allows you to learn by playing (DP3)	6.16	1.156
Using a VR system makes learning more interesting (AU1)	6.26	1.077
I have not been bored using the VR system (AU2)	5.94	1.298
I think using a VR system in the classroom is a good idea (AU3)	6.32	0.994
I would like to use the VR system in the future if I have the opportunity (IU1)	6.25	1.031
I would like to use the VR system to learn anatomy like other topics (IU2)	6.51	0.919

**Table 5:** TAM correlations.

Dimensions	UP	FUP	DP	AU	IU
**UP**		0.473 **	0.690 **	0.730 **	0.693 **
**FUP**			0.444 **	0.484 **	0.395 **
**DP**				0.780 **	0.749 **
**AU**					0.781 **

Note = significant at ** ≤ 0.01.

**Table 6:** Correlations between the TAM and the dimensions that comprise it.

Dimensions	TAM-Total
UP	0.865
FUP	0.680
DP	0.873
AU	0.871
IU	0.821

**Table 7:** Mean scores and standard deviations achieved with the evaluation instrument.

Dimension	M	SD
Technical and aesthetic aspects	4.76	1.370
Ease of navigation and scrolling around the environment	4.77	1.322
Program guide/tutorial	4.76	1.408
Full evaluation	4.76	1.333

**Table 8:** Correlations in the variables “evaluation” and “degree of acceptance of the technology”.

Dimensions	UP	FUP	DP	AU	IU	Total
Technical and aesthetic aspects	0.485 **	0.444 **	0.431 **	0.490 **	0.391 **	0.537 **
Ease of navigation…	0.492 **	0.484 **	0.464 **	0.512 **	0.413 **	0.567 **
Guide/walkthrough…	0.457 **	0.506 **	0.348 **	0.429 **	0.355 **	0.513 **
Total	0.521 **	0.514 **	0.445 **	0.513 **	0.415 **	0.581 **

As we can see, there has been a strong significant relationship at *p* ≤ 0.01 (**) between both variables and between the different dimensions that make up the model, relationships that are positive and of an intermediate nature.

## Data Availability

Data is available at: https://grupotecnologiaeducativa.es/ (accessed on 13 March 2023).
